# P217: Invasive device associated infection rate from 2005 to 2012 in medical intensive care unit at a university hospital in Turkey

**DOI:** 10.1186/2047-2994-2-S1-P217

**Published:** 2013-06-20

**Authors:** B Cinar, H Zengin, Y Gelebek, H Aytac, Z Bastug, Y Sardan, S Unal

**Affiliations:** 1 İnfection Control Unit, Hacettepe University Adult Hospital, Ankara, Turkey; 2 İnfection Control Unit, Hacettepe University Oncology Hospital, Ankara, Turkey; 3 Guven Hospital, Ankara, Turkey

## Introduction

Invasive Device Associated Infection (IDAI) rate in healthcare facilities is one of the important quality indicators. IDAI rate should be monitored and necessary precautions should be implemented. Surveillance for IDAI has been started in 2001 at Hacettepe University (H.U.) intensive care units (ICU). When compared the year 2001 to 2005 surveillance results with other reports it was seen as higher than developed countries. In 2006 Ventilator-associated pneumonia (VAP) rate was doubled than 90 percentiles therefore a several interventions were conducted to decrease VAP and Central Line-Associated Bloodstream Infection (CLABSI) rate in all ICUs since 2007.

## Objectives

Aim of the study was to evaluate IDAI rates from 2005 to 2012 in medical ICUs at H. U. Adult hospital.

## Methods

A prospective, active surveillance has been employed in medical intensive care units with using National Healthcare Safety Network (NHSN) method. The IDAI defined according to “Centers for Disease Control and Prevention” criteria and computed infection rate compared NHSN numbers/scores. Improvement works began for the rate over than 50.percentile; such as written procedures, implementing preventive measure/precautions, continuing education, audit and observations were performed. New database program established in 2005 and surveillance data entered to this program. Results were summarized every 3 months and shared with intensive care units employees and discussed their opinions/suggestions for improvement.

## Results

The VAP rate which was 30.6/1000 catheter day in year 2006 was decreased in to half in 2007; after that continued as 10/1000 and below catheter day. Similarly CLABSI rate was lowered from 18.5/1000 to 8/1000 catheter day and continued as below 8/1000 catheter day. During these years urinary tract infection rates were not changed. When compared these rates since 2007 with other countries it was less than 50 percentile.

## Conclusion

It was seen that at the hospital in limited resource country, IDAI rates can be decreased when infection control measures implemented and complied as well as surveillance results regularly followed/monitored.

## Disclosure of interest

None declared

